# Prognostic value of the lactate-albumin ratio in trauma: A comparison with shock index and injury severity score

**DOI:** 10.1371/journal.pone.0326367

**Published:** 2025-07-01

**Authors:** Jonghan Park, Wook Tae Yang, Seok-Ran Yeom, Sung-Wook Park, Young Mo Cho, Won Ung Tae, Hyuk Jin Choi, Mahnjeong Ha, Seunghan Yu, Up Huh, Dongman Ryu, Chanhee Song, Il Jae Wang

**Affiliations:** 1 Department of Emergency Medicine, Pusan National University School of Medicine and Biomedical Research Institute, Pusan National University Hospital, Busan, Republic of Korea; 2 Department of Neurosurgery, Pusan National University School of Medicine and Biomedical Research Institute, Pusan National University Hospital, Busan, Republic of Korea; 3 Department of Thoracic and Cardiovascular Surgery, Pusan National University School of Medicine and Biomedical Research Institute, Pusan National University Hospital, Busan, Republic of Korea; 4 Medical Research Institute, Pusan National University, Busan, Republic of Korea; Azienda Ospedaliero Universitaria Careggi, ITALY

## Abstract

This study aimed to assess the predictive value of lactate-to-albumin ratio (LAR) for in-hospital mortality and massive transfusion (MT) in severe trauma cases. This retrospective, observational, single-center study included patients who presented to a trauma center between 2016 and 2022. The primary outcome was in-hospital mortality, and the secondary outcome was MT. Logistic regression analysis was performed to determine whether LAR was an independent risk factor. The area under the receiver operating characteristic (AUROC) curve was calculated to evaluate the predictive value of LAR. In total, 5,304 patients were included. Logistic regression analysis identified LAR as an independent risk factor for in-hospital mortality. The AUROCs for predicting in-hospital mortality using LAR, Injury Severity Score (ISS), and Shock Index (SI) were 0.74 (95% confidence interval [CI], 0.73–0.75), 0.78 (95% CI, 0.77–0.779), and 0.51 (95% CI, 0.49–0.52), respectively. LAR was statistically significant compared with SI (p < 0.0001; 95% CI, 0.194–0.276) and similar to ISS (p = 0.0039; 95% CI, 0.0115–0.0604). LAR (0.84; 95% CI, 0.83–0.85) demonstrated superior predictive power for MT compared with SI (0.68; 95% CI, 0.67–0.70) and ISS (0.79; 95% CI, 0.78–0.80). LAR demonstrates a superior predictive ability for in-hospital mortality and MT.

## Introduction

Trauma remains a major cause of morbidity and mortality worldwide; therefore, ongoing efforts are required to improve risk stratification and prognosis in trauma care [1]. Early identification of patients at a high risk of adverse events, such as in-hospital mortality, is important for timely intervention and resource allocation [2].

Existing prognostic scoring systems, such as the Injury Severity Score (ISS), Trauma and Injury Severity Score (TRISS), and Revised Trauma Score (RTS), are widely used to assess trauma severity and predict patient outcomes [3–5]. Although these scoring systems provide valuable clinical information, their calculation can be complex and time-consuming in emergency settings. In contrast, lactate is a single biomarker that can be rapidly measured and offers the advantage of reflecting a patient’s metabolic status and severity of shock with a single value, facilitating prompt clinical decision-making [6].

Serum lactate concentration has been used as a parameter of tissue hypoperfusion and anaerobic metabolism in patients who are critically ill, including those with traumatic injuries [7–9]. Elevated lactate levels are associated with increased mortality, reflecting the severity of shock and degree of tissue damage [10,11]. However, lactate measurements alone cannot provide a comprehensive assessment of a patient’s metabolic status [7].

Albumin, a negative acute-phase reactant synthesized in the liver, is an indicator of nutritional status and systemic inflammation [12]. By integrating lactate and albumin measurements, lactate-to-albumin ratio (LAR) may provide additional prognostic information beyond that provided by lactate alone. Although lactate and albumin levels have been studied individually as predictors of prognosis in patients with trauma [13–15], studies on the use of LAR in patients with trauma are limited.

This study evaluates LAR’s predictive value for in-hospital mortality and massive transfusion (MT) in trauma patients, directly comparing its performance to SI and ISS. We hypothesize that LAR will outperform traditional scores in risk stratification.

## Materials and methods

### Study design

This retrospective, single-center study evaluated the predictive power of LAR for mortality in patients with trauma using the medical records of patients admitted to Pusan National University Hospital (PNUH). This study was conducted at the Trauma Center of PNUH. This center is one of the largest trauma centers in Korea, with 1,400 beds, and annually accommodates approximately 1,000 patients with an Injury Severity Score (ISS) of ≥15. This was a retrospective study using the medical records of patients admitted to our hospital, in which patient information was anonymized, and data were accessed for study from May 20 to June 20, 2024. The clinical trial was approved by the PNUH Institutional Review Board for exemption from the requirement for informed consent (approval number: 2405-017-139).

### Study population

The study population consisted of consecutive patients who presented to our hospital’s trauma center between 2016 and 2022, and patients who met the following criteria were excluded from this study: (1) patients aged <16 years, (2) patients with cardiac arrest upon arrival at the trauma center, and (3) patients with missing initial albumin and lactate levels.

### Data collection

Data from the study population, including age, sex, mechanism of injury, vital signs (systolic blood pressure [SBP], diastolic blood pressure [DBP], heart rate [HR]) at trauma center arrival, injury severity score (ISS), presence or absence of blood transfusion, massive transfusion (MT), in-hospital mortality, and 24-h mortality, were used in this study. Laboratory results, such as prothrombin time international normalized ratio (PT INR), activated partial thromboplastin time (aPTT), hemoglobin level, platelet count, and lactate, albumin, LAR, aspartate transaminase (AST), and creatine levels, were collected. Blood tests were taken as soon as the patient arrived at the emergency room.

The SI is defined as the ratio of heart rate to systolic blood pressure. The ISS is an anatomical scoring system derived from the Abbreviated Injury Scale (AIS). It is calculated as the sum of the squares of the highest AIS code in each of the 3 most severely injured ISS body regions. At our trauma center, two trauma surgeons and two trained nurses, certified by the Association for the Advancement of Automotive Medicine, assessed the AIS and entered the results into the trauma registry. The ISS value was calculated based on the input results.

### Outcome measures

The primary outcome was in-hospital mortality, and the secondary outcome was MT. MT was defined as the transfusion of >10 units of packed red blood cells within 24 h [16].

### Statistical analyses

The variables are presented as frequencies (N) and percentages (%) for categorical variables and as means, standard deviations, or medians for continuous variables. Categorical variables were assessed using the chi-squared test, and continuous variables were evaluated using the Student T-test and Mann–Whitney U test. The Kolmogorov–Smirnov test was performed to assess the normality of variables. Multiple logistic regression analysis was performed to evaluate the independent predictors of in-hospital mortality and MT. A receiver operating characteristic (ROC) curve was created, and the area under the ROC curve (AUROC) was calculated to compare the predictive power of the variables. The optimal cutoff value was calculated using the Youden index. Statistical significance was defined as a two-sided p-value <0.05, and all statistical analyses were performed using MedCalc version 22.007 (MedCalc Inc., Mariakerke, Belgium).

## Results

### Patient characteristics

**Fig 1:**
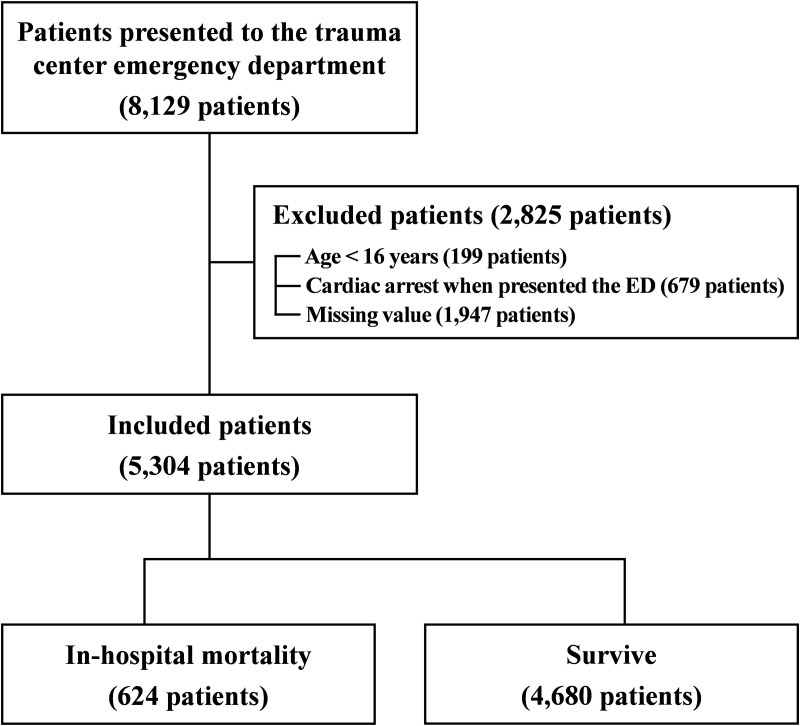
Procedure of patient selection for this study.

A total of 8,129 patients who presented to trauma centers were considered for this study. In total, 2,825 patients met the exclusion criteria. Their details were as follows: (1) age < 16 years (199 patients), (2) cardiac arrest upon arrival at the trauma center (679 patients), and (3) missing lactate and albumin levels (1,947 patients). Finally, 5,304 patients were included in this study. Trauma sites were classified using AIS scores ≥2. Head/neck injuries were the most prevalent (55.02%, n = 2,918), followed by thoracic (51.28%, n = 2,720), extremity (51.09%, n = 2,710), abdominal/pelvic (42.38%, n = 2,248), facial (17.29%, n = 917), and external injuries (0.36%, n = 19). Of the 5,304 patients, 52.66% (n = 2,793)were transported directly via emergency medical services (EMS), while 47.34% (n = 2,511) were transferred from other hospitals. The mean time from injury to hospital arrival for EMS-transported patients was 63.07 minutes (median: 45 minutes). Of these, in-hospital mortality was observed in 624 patients. The details are shown in Fig 1. The mean age of the patients was 57.38 years, and 75.6% were male. Traffic accidents (TAs) and falls accounted for 48.45% and 28.24% of the injury mechanisms, respectively. Slip down, penetrating, object down, and others accounted for <10% of the mechanisms of injury. The mean ISS and in-hospital mortality rates were 20.41 and 6.97%, respectively. In total, 2,814 (53%) patients received transfusions, and 487 (9.18%) patients received MT. The mean values of lactate, albumin, and LAR were 3.37, 3.91, and 0.96, respectively (Table 1).

**Table 1:** Comparison of variables of the in-hospital mortality and survival groups.

Variables	Total (n = 5,304)	In-hospital mortality (n = 624)	Survival (n = 4,680)	P-value
Age (y), mean (SD)	57.38 (18.04)	65.42 (17.52)	56.31 (17.84)	<0.0001
Sex, n (%)				0.0915
Male	4,012 (75.6)	455 (72.9)	3,557 (76.0)	
Female	1,292 (24.4)	169 (27.1)	1,123 (24.0)	
Mechanism of injury, n (%)				<0.0001
TA	2,570 (48.45)	310 (49.68)	2,260 (48.29)	
Slip down	322 (6.07)	38 (6.09)	284 (6.07)	
Penetrating	374 (7.05)	9 (1.44)	365 (7.80)	
Object blunt	406 (7.65)	27 (4.33)	379 (8.10)	
Fall down	1,498 (28.24)	203 (32.53)	1,295 (27.67)	
Others	134 (2.53)	37 (5.93)	97 (2.07)	
SBP, mean (SD)	115.49 (38.32)	107.42 (58.75)	116.57 (34.57)	<0.0001
DBP, mean (SD)	68.44 (28.24)	59.31 (40.24)	69.66 (26.0)	<0.0001
HR, mean (SD)	91.91 (22.09)	97.85 (28.27)	91.12 (20.94)	<0.0001
GCS, mean (SD)	12.46 (4.02)	6.96(4.37)	13.20 (3.35)	<0.0001
ISS, mean (SD)	20.41 (11.48)	29.97 (11.15)	19.14 (10.91)	<0.0001
In-hospital 24-h mortality, n (%)				<0.0001
No	4,952 (93.03)	272 (43.59)	4,680 (100)	
Yes	352 (6.97)	352 (56.41)	–	
Blood transfusion, n (%)				<0.0001
No	2,490 (46.95)	86 (13.78)	2,404 (51.37)	
Yes	2,814 (53.05)	538 (86.22)	2,276 (48.63)	
Massive blood transfusion, n (%)				<0.0001
No	4,817 (90.82)	398 (63.78)	4,419 (94.42)	
Yes	487(9.18)	226 (36.22)	261 (5.58)	
PT INR, mean (SD)	1.16 (0.45)	1.54 (1.14)	1.11 (0.19)	<0.0001
aPTT time, mean (SD)	28.09 (12.45)	41.42 (28.36)	26.37 (6.73)	<0.0001
Hemoglobin, mean (SD)	13.10 (2.75)	11.79 (3.17)	13.28 (2.64)	<0.0001
Platelet, mean (SD)	220.80 (73.91)	185.61 (77.62)	225.47 (72.14)	<0.0001
Lactate, mean (SD)	3.37 (2.72)	5.54 (3.95)	3.08 (2.37)	<0.0001
Albumin, mean (SD)	3.91 (0.68)	3.38 (0.84)	3.99 (0.62)	<0.0001
LAR, mean (SD)	0.96 (0.015)	1.94 (1.95)	0.83 (0.79)	<0.0001
AST, mean (SD)	112.22 (204.62)	150.29 (278.59)	107.15 (192.10)	<0.0001
Creatine, mean (SD)	0.95 (0.60)	1.14 (0.91)	0.92 (0.54)	<0.0001

TA, traffic accident; SBP, systolic blood pressure; DBP, diastolic blood pressure; HR, heart rate; GCS, Glasgow Coma Scale; ISS, Injury Severity Score; PT INR, prothrombin international normalized ratio; aPTT, activated partial thromboplastin time; LAR, lactate-to-albumin ratio; AST, aspartate transaminase.

### Comparison of in-hospital mortality and survival groups

The in-hospital mortality and survival groups were compared. The mean age of the in-hospital mortality group was 65.42 years, which was 9.11 years higher than that of the survival group (56.31 years). The mean values of SBP and DBP were lower, and HR was higher in the in-hospital mortality group than those in the survival group; and GCS was lower and ISS was higher in the in-hospital mortality group than those in the survival group. The in-hospital mortality group had a higher MT rate than the survival group. Lactate levels and LAR in the in-hospital mortality group were significantly higher than those in the survival group. Table 1 shows the comparison of variables of the in-hospital mortality and survival groups.

### Univariate and multivariate logistic regression analyses

We performed univariate and multivariate logistic regression analyses of risk factors for in-hospital mortality. Collinearity was identified using the variance inflation factor (VIF) method, and VIF of ≥10 was considered to have collinearity. Variables with p < 0.05 in the univariate analysis were included in the multivariate regression analysis.

Univariate logistic regression analysis revealed that shock index (SI), ISS, PT INR, LAR, and creatine level were risk factors for in-hospital mortality. ISS, PT INR, LAR, and creatine levels were correlated with outcomes in the multivariate logistic regression analysis. In the multivariate analysis, ISS (odds ratio [OR], 1.04; 95% confidence interval [CI], 1.03–1.05); p < 0.0001), PT INR (OR, 2.93; 95% CI, 1.96–4.36; p < 0.0001), LAR (OR, 1.19; 95% CI, 1.07–1.32; p = 0.0014), and creatine level (OR, 1.34; 95% CI, 1.18–1.52; p < 0.0001) were independent predictors of in-hospital mortality (Table 2).

**Table 2:** Univariate and multivariate logistic regression analyses of risk factors for in-hospital mortality.

Variable	Univariate analysis		Multivariate analysis	
	OR (95% CI)	P-value	OR (95% CI)	P-value
Age	1.03 (1.03–1.04)	0.09		
Male (vs. female)	1.18 (0.97–1.42)	0.095		
Mechanism of injury				
TA	Ref		Ref	
Slip down	0.98 (0.68–1.40)	0.89	1.07 (0.69–1.66)	0.78
Penetrating	0.18 (0.09–0.35)	<0.0001	0.59 (0.26–1.32)	0.20
Object blunt	0.52 (0.35–0.78)	<0.0001	0.97 (0.60–1.59)	0.92
Fall down	1.14 (0.95–1.38)	0.17	1.26 (0.99–1.60)	0.06
Others	2.78 (1.87–4.14)	<0.0001	1.40 (0.86–2.29)	0.18
GCS	0.73 (0.71–0.74)	<0.0001	0.77 (0.75–0.79)	<0.0001
SI	1.08 (1.07–1.09)	<0.0001	1.00 (1.00–1.01)	0.51
ISS	1.02 (1.0–1.04)	<0.0001	1.04 (1.03–1.05)	<0.0001
PT INR	24.9 (17.70–35.04)	<0.0001	2.93 (1.96–4.36)	<0.0001
Hemoglobin	0.84 (0.82–0.87)	0.08		
Platelet	0.99 (0.99–0.99)	0.0001	1.00 (0.99–1.00)	<0.0001
LAR	1.99 (1.85–2.14)	<0.0001	1.19 (1.07–1.32)	0.0014
AST	1.00 (1.00–1.00)	<0.0001	1.00 (1.00–1.00)	0.36
Creatine	1.46 (1.31–1.63)	<0.0001	1.34 (1.18–1.52)	<0.0001

OR, odds ratio; CI, confidence interval; TA, traffic accident; GCS, Glasgow Coma Scale; SI, Shock Index; ISS, Injury Severity Score; PT INR, prothrombin international normalized ratio; LAR, lactate-to-albumin ratio; AST, aspartate transaminase.

### Receiver operating characteristic curve analysis

**Fig 2:**
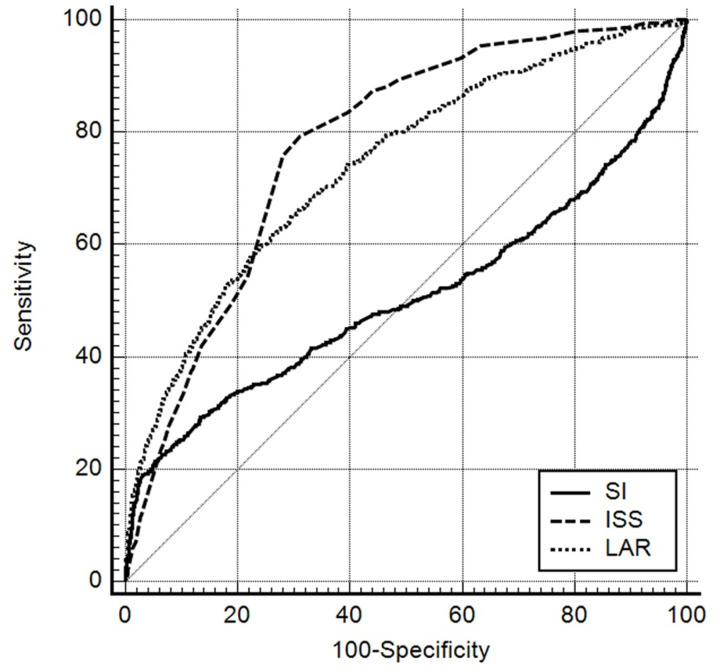
Receiver operating characteristic curves of Shock Index, Injury Severity Score, and lactate-to-albumin ratio for predicting in-hospital mortality.

**Fig 3:**
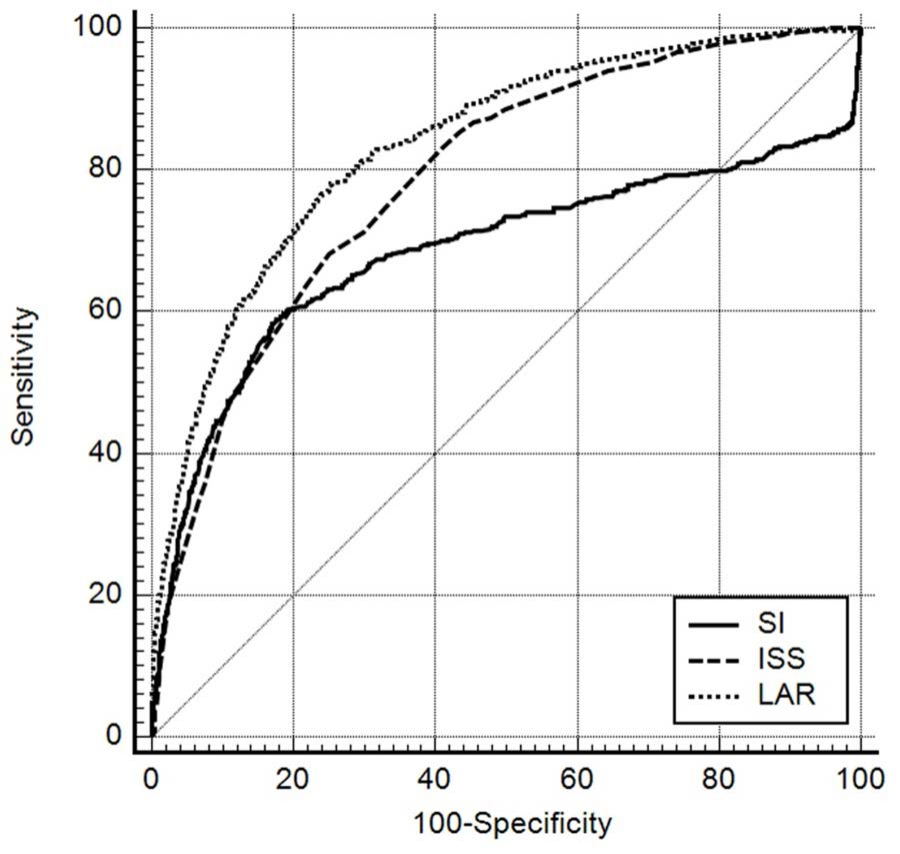
Receiver operating characteristic curves of Shock Index, Injury Severity Score, and lactate-to-albumin ratio for predicting massive transfusion.

ROC curve analysis was performed to evaluate the predictive power of in-hospital mortality and MT. The AUROC values for SI, ISS, and LAR in predicting in-hospital mortality were 0.51 (95% CI, 0.49–0.52), 0.78 (95% CI, 0.77–0.779), and 0.74 (95% CI, 0.73–0.75), respectively. For predicting MT, the AUROC values for SI, ISS, and LAR were 0.68 (95% CI, 0.67–0.70), 0.79 (95% CI, 0.78–0.80), and 0.84 (95% CI, 0.83–0.85), respectively (Table 3 and Figs 2–3).

**Table 3:** Receiver operating characteristic curve analysis results for in-hospital mortality and MT.

	AUROC (95% CI)	Cutoff point	Sensitivity	Specificity	PPV	NPV
In-hospital mortality						
SI	0.51 (0.49–0.52)	0.43	21.47	94.66	34.7	90
ISS	0.78 (0.77–0.79)	22	79.33	68.78	25.3	96.1
LAR	0.74 (0.73–0.75)	1.06	58.33	77.46	25.5	93.3
MT						
SI	0.68 (0.67–0.70)	1.05	58.93	81.86	24.7	95.2
ISS	0.79 (0.78–0.80)	25	68.17	74.84	21.5	95.9
LAR	0.84 (0.83–0.85)	1.02	76.39	76.4	24.4	97

AUROC, area under the ROC curve; CI, confidence interval; PPV, positive predictive value; NPV, negative predictive value; SI, Shock Index; ISS, Injury Severity Score; LAR, lactate-to-albumin ratio.

## Discussion

The main findings of this study demonstrate that LAR is a valuable prognostic indicator for trauma patients. In the multivariate logistic regression analysis, LAR was independently associated with in-hospital mortality in trauma patients. Analysis results using AUROC values, LAR was useful for predicting in-hospital mortality and MT. In predicting in-hospital mortality, the predictive power of LAR (0.74) was superior to SI (0.51) and comparable to that of ISS (0.78). In predicting MT, the predictive power of LAR (0.84) was superior to SI (0.68) and ISS (0.79).

Lactate, a byproduct of anaerobic metabolism generated under hypoxic conditions, is widely recognized as an indicator of inadequate peripheral tissue perfusion. Elevated serum lactate levels reflect microcirculatory dysfunction and have been positively correlated with increased mortality across various clinical scenarios [17–20]. In trauma, elevated serum lactate is not only a marker of tissue hypoperfusion but also serves as a sensitive and early indicator of occult shock, even when vital signs appear normal [21,22]. In 2023, Arslan et al. demonstrated that elevated lactate levels in trauma patients were significantly associated with increased mortality [23]. However, the prognostic value of lactate alone may be limited in patients with comorbidities such as hepatic or renal impairment, diabetes mellitus, metformin use, malignancy, or chronic alcohol consumption, which can alter lactate metabolism [24–27]. Albumin, an indicator of nutritional status and chronic inflammation, may complement lactate by reflecting a patient’s baseline physiological reserve. Consequently, the LAR has been investigated as a prognostic marker in various critically ill populations, including those with sepsis, cardiac arrest, and other acute conditions [28–33].

Recent studies have explored the prognostic utility of the LAR in trauma patients. Wang et al. (2022) demonstrated that LAR was associated with in-hospital mortality in patients with severe traumatic brain injury (TBI) [34], and Lee et al. similarly reported its association with both in-hospital mortality and MT in TBI patients [35]. A consistent observation across these studies was that LAR levels were significantly elevated in non-survivors and served as an independent predictor of mortality. In our cohort, a substantial proportion of patients had concomitant TBI, aligning with these findings. Beyond TBI, Dudoignon et al. identified LAR as a reliable predictor of 28-day mortality in patients with severe burn injuries [36]. In 2022, Sangwoo et al. reported that LAR was independently associated with multiple organ failure and 30-day mortality in 348 patients with severe trauma (ISS ≥ 16), showing prognostic performance comparable to Acute Physiology and Chronic Health Evaluation II (APACHE II), ISS, and SI [37]. Similarly, Arslan et al., in a study of 176 patients with multiple trauma, confirmed the prognostic value of LAR as an independent predictor of mortality [38]. These results were consistent with ours. While these studies support the clinical relevance of LAR, their relatively small sample sizes (n = 348 and n = 176) limit generalizability. In contrast, our study, involving a large cohort of 5,304 trauma patients, provides robust evidence for the prognostic value of LAR. Notably, our findings extend previous research by confirming its association with the need for MT, underscoring LAR’s potential utility in guiding timely transfusion decisions in trauma care.

We hypothesized that LAR would serve as a superior predictor of in-hospital mortality and MT compared to conventional scoring systems. This hypothesis assumes that LAR combines tissue hypoperfusion and metabolic resilience. Although established tools such as the SI and ISS are widely used in clinical practice, each has limitations. SI lacks specificity in patients with chronic conditions. Because SI is based on vital signs, it may fail to capture underlying metabolic or inflammatory stress, particularly in cases of compensated shock. ISS assesses anatomical trauma severity but requires imaging and detailed scoring, which may delay timely clinical decisions. In contrast, LAR offers a real-time, integrative assessment of a patient’s physiological status through a readily available blood test. Our findings support this hypothesis. These results highlight the potential of LAR as a practical and rapid tool for identifying critically ill trauma patients and facilitating prompt, informed decision-making during resuscitation.

This study had some limitations. First, because this study was conducted at a single center, generalizations to other institutions are limited. Second, because this was a retrospective study, there is a possibility of selection bias. Third, there were no data on prehospital treatment, which may have led to changes in the initial measurements. Finally, there may have been differences in treatment methods, such as albumin replacement and the total amount of fluid administered, which may have resulted in differences in patient prognosis. Future studies should consider prospective multicenter investigations to mitigate selection bias and enhance generalizability.

## Conclusion

Our findings show that LAR is an independent risk factor for in-hospital mortality and a useful indicator for predicting both in-hospital mortality and MT in patients with trauma.

## Supporting information

S1 Data Trauma patients data (2016-2022) (XLSX)
